# Dimethyl Fumarate Triggers the Antioxidant Defense System in Human Retinal Endothelial Cells through Nrf2 Activation

**DOI:** 10.3390/antiox11101924

**Published:** 2022-09-28

**Authors:** Federico Manai, Marialaura Amadio

**Affiliations:** 1 Department of Biology and Biotechnology “L. Spallanzani”, University of Pavia, 27100 Pavia, Italy; 2 Department of Drug Sciences, Section of Pharmacology, University of Pavia, 27100 Pavia, Italy

**Keywords:** dimethyl fumarate, Nrf2, HREC, cytoprotection, antioxidant, repurposing

## Abstract

Dimethyl fumarate (DMF) is a well-known activator of Nrf2 (NF-E2-related factor 2), used in the treatment of psoriasis and multiple sclerosis. The mechanism of action consists in the modification of the cysteine residues on the Nrf2-inhibitor Keap1, thus leading to the dissociation of these two proteins and the consequent activation of Nrf2. Considering the paucity of evidence of DMF effects in the context of retinal endothelium, this *in vitro* study investigated the role of DMF in human retinal endothelial cells (HREC). Here, we show for the first time in HREC that DMF activates the Nrf2 pathway, thus leading to an increase in HO-1 protein levels and a decrease in intracellular ROS levels. Furthermore, this molecule also shows beneficial properties in a model of hyperglucose stress, exerting cytoprotective prosurvival effects. The overall collected results suggest that DMF-mediated activation of the Nrf2 pathway may also be a promising strategy in ocular diseases characterized by oxidative stress. This study opens a new perspective on DMF and suggests its potential repositioning in a broader therapeutical context.

## 1. Introduction

The Nrf2 (NF-E2-related factor 2) signaling pathway is a major and evolutionarily conserved defensive system whose main function is to orchestrate the cellular response to oxidative stress and regulate the basal and inducible expression of many cytoprotective genes. Nrf2 contributes to maintaining the cellular reduction–oxidation homeostasis and a youthful phenotype, also playing a primary role in cell response against pro-oxidant and proinflammatory stress [1]. 

Consequently, *Nrf2* knockout in animals increases their susceptibility to a wide range of chemical toxicity and disease conditions associated with oxidative stress [2,3,4,5]. Nrf2 alterations have been found in several chronic pathologies, including neurodegenerative diseases, ischemia, atherosclerosis, and asthma [6,7,8]. 

Pharmacologic activation of Nrf2 has thus emerged as an attractive strategy for many pathologic conditions [9]. In agreement, in the last years, increasing research efforts have been devoted to identifying novel molecules able to activate the Nrf2 pathway. In many cases, pharmacologic activation of Nrf2 is obtained by the dissociation of Nrf2 from its negative regulator Keap1 (Kelch-like ECH-associated protein 1). Under homeostatic conditions, Keap1 maintains Nrf2 sequestered and inactive in the cytosol and directs it to ubiquitination and proteasomal degradation with a fast turnover [10]. Inhibition of Keap1 by endogenous and exogenous molecules, including reactive oxygen species (ROS), prevents degradation of Nrf2 by its dissociation from the suppressor Keap1. Once free, Nrf2 translocates into the nucleus and binds to antioxidant response elements (ARE) in the DNA, inducing the transcription of an array of antioxidant and detoxifying enzyme genes, such as *Heme Oxygenase-1* (*HO-1*) [3,11].

The majority of Nrf2 activators is represented by the protein–protein interaction inhibitors (PPI), molecules mainly able to modify cysteine residues of Keap1, leading to dissociation from Nrf2 and its consequent activation [12]. The best-known PPI is dimethyl fumarate (DMF), a fumaric acid ester that can oxidize sulfhydryl (-SH) groups of Keap1, finally activating Nrf2 [13]. Both *in vitro* and *in vivo *studies extensively demonstrated that DMF, via Nrf2 pathway, exerts cytoprotective effects in neurons and glial cells under proinflammatory and pro-onxidant conditions [14,15]. In virtue of its clinical benefits, DMF has been approved and is currently used for the treatment of psoriasis and relapsing-remitting multiple sclerosis [16,17]. 

However, ongoing preclinical and clinical studies suggest that DMF potential therapeutic use may be wider than expected [18]. The evidence from studies of animal models of eye diseases shows that DMF also displays beneficial effects at the ocular level. For instance, a recent *in vivo* study showed that DMF has survival-promoting effects in retinal ganglion cells after optic nerve crush, possibly through the Nrf2/HO-1 pathway [19]. A case report describes a successful therapeutic attempt with DMF for macular edema, an ocular pathology characterized by vascular dysfunction and inflammation [20]. In agreement, DMF exerts benefits at both the vascular and neuronal level by activation of Nrf2/HO-1 pathway, as shown in animal models of intracerebral hemorrhage [21,22].

The data from studies suggest that Nrf2/HO-1 plays a key role in protecting the endothelium from ROS-related injuries [23]. Coherently, some *in vitro* studies show that DMF counteracts inflammation in endothelial cells exposed to various cytokines/chemicals/toxic stimuli [24,25]. 

Based on these premises, it is presumable that DMF may activate the Nrf2/HO-1 pathway and exert beneficial effects in the vascular endothelium of the retina; however, these hypotheses have never been tested in such a cellular context. With the aim to fill this research gap and to further explore the therapeutic potentiality of DMF, here we evaluated in an *in vitro* model based on human retinal endothelial cells (HREC) the DMF capability to activate Nrf2 and, downstream, the expression of HO-1, one of the most important antioxidant and anti-inflammatory enzymes in vascular endothelium [26,27]. Alterations of retinal endothelial cells are a key pathogenic factor of many ocular diseases, including diabetic retinopathy (DR), a common complication of diabetes affecting both neural and vascular districts of the retina, leading to progressive visual impairment and, in the worst cases, sight loss [28,29]. For this reason, we also tested *in vitro* DMF’s potential cytoprotective effects against a DR-related insult. Our findings in HREC show that DMF is optimally tolerated and that it can activate the Nrf2/HO-1 pathway and confer cytoprotection under high glucose conditions.

Together with the most recent evidence of DMF effects at the ocular level, our findings open a new perspective for the repositioning of DMF in eye pathologies involving alterations of the endothelium and, more extensively, for ocular diseases characterized by oxidative stress and inflammation.

## 2. Materials and Methods

### 2.1. Cell Culture and Treatments

Human retinal endothelial cells (HREC) used in this study were obtained from Innoprot (Derio, Bizkaia, Spain). Cells were grown in Endothelial Cell Medium supplemented with 5% fetal bovine serum (FBS), 1% of endothelial cell grown supplements, and 1% penicillin/streptomycin. All reagents were provided by Innoprot. The incubator settings were 37 °C for temperature and 5% CO_2_ in humidified atmosphere. Cell passage number less than 15 was used in the study. Approximately 125,000 cells per well were seeded on 24-well plates and incubated for 24 h to reach confluence. Dimethyl fumarate (DMF) was introduced to the cells by removing the old culture medium and adding fresh medium containing DMF compound diluted in dimethyl sulfoxide (DMSO) at different concentrations, according to the experimental setting. The final concentration of DMSO in the experiments did not exceed the 0.025% of the final volume.

For high glucose experiments, HREC were exposed to glucose [30 mM and 40 mM] or mannitol [30 mM] for increasing times (24 h, 48 h, 72 h; 6 days); control HREC were exposed for the same time to normal glucose [5 mM]. 

### 2.2. Protein Extraction and Western Blotting

After treatments, the cells were subjected to the following published protocol [30]. Specifically, cells were washed twice with calcium/magnesium-free Dulbecco’s phosphate buffered saline (PBS, MilliporeSigma, Burlington, MA, USA) and lysed in 75 µL of Mammalian Protein Extraction Reagent (M-PER, Thermo Fisher Scientific, Waltham, MA, USA). The M-PER reagent was left on the cells for 3 min, the wells were scraped on ice and protein lysates collected. The lysates were centrifuged at 13,000× *g* for 15 min at 4 °C and the supernatants were stored at −70 °C until analysis. The protein concentrations of HREC cell lysates were measured using the Bradford protein assay method. Samples containing 25 µg of protein were run into 12% SDS-PAGE gels. The protein bands were then transferred onto nitrocellulose membranes (GE Healthcare, Chicago, IL, USA) in an overnight wet blot at 4 °C. Ponceau S (MilliporeSigma) staining was done on the membranes to confirm good protein transfer.

The membranes were cut into two parts, above the 70 kDa and 25 kDa bands, and blocked as follows: upper part in 5% BSA, 0.1% Tween^®^ 20 (MilliporeSigma)—phosphate-buffered saline (T-PBS) solution; middle part in 3% milk, 0.3% T-PBS solution; lower part in 3% milk, 0.1% T-PBS solution, for 1.5 h (upper and middle parts) or 2 h (lower part) at RT. The upper parts were then incubated overnight at +4 °C with Nrf2 primary antibody (Novus biologicals LLC, Centennial, CO, USA) (1:1000 in 5% BSA, 0.1% T-PBS). The lower part of the membrane was incubated with primary antibodies for HO-1 (Novus biologicals LLC), α-tubulin (MilliporeSigma) (1:8000 in 1% milk, 0.05% T-PBS; for 0.5 h at RT), or β-actin (Cell Signaling Technology, Danvers, MA, USA) (1:6000 in 5% milk, 0.05% T-PBS; horseradish peroxidase-conjugated). After that, the membranes were washed for 3 × 5 min with their respective washing buffers. Horseradish peroxidase-conjugated anti-mouse (MilliporeSigma) or anti-rabbit (Thermo Fisher Scientific) IgG secondary antibodies were then incubated at RT as follows: anti-mouse 1:10,000 in 1% milk, 0.05% T-PBS (α-tubulin) for 0.5 h, or anti-rabbit 1:10,000 in 5%BSA, 0.1% T-PBS (Nrf2, HO-1) for 2 h. The membranes were then washed 3 × 5 min. Immobilon Western Chemiluminescent HRP Substrate (MilliporeSigma) was applied for 5 min and the protein bands were detected using ImageQuant RT ECL Imager (GE Healthcare). The results were quantified using the ImageJ program (https://imagej.nih.gov/ij/, accessed on 30 May 2022).

### 2.3. RNA Extraction, Retro-Transcription, and Real-Time Quantitative PCR

Total RNA was extracted from HREC by the Direct-zol RNA MiniPrep Kit (Zymo Research, Irvine, CA, USA) and subjected to reverse transcription by the QuantiTect Reverse Transcription Kit (Qiagen, Hilden, Germany) following standard procedures. Real-time quantitative PCR (RT-qPCR) amplifications were carried out using the QuantiTect SYBR Green PCR Kit (Qiagen) and the Lightcycler instrument (Roche, Basel, Switzerland), with the following primers previously validated [31]: 

*Nrf2* (Gene ID: 4780): 5′- TTCTGTTGCTCAGGTAGCCCC -3′ (upstream) and

5′- TCAGTTTGGCTTCTGGACTTGG -3′ (downstream);

*HO-1* (Gene ID: 3162): 5′- AGCAACAAAGTGCAAGATTCTGC -3′ (upstream) and

5′- CAGCATGCCTGCATTCACATG -3′ (downstream);

*GAPDH* (Gene ID: 2596): 5′- CAGCAAGAGCACAAGAGGAAG -3′ (upstream) and

5′- CAACTGTGAGGAGGGGAGATT -3′ (downstream). 

*GAPDH* mRNA was the reference on which all the values were normalized, due to its substantial stability in our experimental conditions, as in most cases in the literature. A 2^−ΔΔCt^ method was used for the mRNA quantification.

### 2.4. Cell Viability Assay

HREC were plated 20,000/well in a 96-well plate, and cell viability was determined by PrestoBlue^®^ assay (Invitrogen, Waltham, MA, USA), following the manufacturer’s instructions and as previously reported [31]. After treatments, cells were loaded for 30 min with PrestoBlue^®^ reagent prior to assay readout. Fluorescence was measured by the Synergy HT multidetection microplate reader (BioTek, Winooski, VT, USA), with excitation and emission wavelengths of 530 and 590 nm, respectively. The results are expressed as a percentage of the fluorescence of the samples in comparison to control (100%).

### 2.5. Immunofluorescence Assay

As previously reported [31], HREC were seeded (25,000 cells) on glass coverslips and cultivated 48 h until they reached confluence. After DMF treatment (i.e., 10 and 50 µM for 6 h), cells were washed twice with PBS and fixed using paraformaldehyde 4% for 20 min at RT. Then, the coverslips were incubated 45 min with anti-Nrf2 primary antibody (diluted 1:30 in 5% non-fat milk in T-TBS). Then, after three washes with T-TBS, coverslips were incubated 30 min in the dark with a species-specific secondary antibody conjugated with AlexaFluor488 (diluted 1:60 in 5% non-fat milk in T-TBS). Lastly, after three washes with T-TBS, coverslips were mounted onto glass slides using ProLong Gold Antifade Reagent with DAPI (Invitrogen, Waltham, MA, USA). Images were obtained using the confocal microscope Leica TCS SP8 DLS (Leica, Wetzelar, Germany) at 60× magnification. 

### 2.6. Flow Cytometry

HREC were seeded (100,000 cells) in a multiwell-24. After DMF treatment (i.e., 10 and 50 µM for 6 h), cells were washed twice with PBS and collected after trypsinization. Then, intracellular ROS levels were analyzed using Muse Oxidative Stress Kit (Luminex, Austin, TX, USA) according to the manufacturer’s instructions. Briefly, the pellet was resuspended in 10 µL of Assay Buffer 1× and 190 µL of prediluted Oxidative Stress reagent (1:800). Cells were then incubated at 37 °C for 30 min before the analysis. 

### 2.7. Statistics

For the statistical analyses, the GraphPad InStat program (GraphPad software, San Diego, CA, USA) was used. Data were subjected to the analysis of variance (ANOVA) and followed, when significant, by an appropriate post-hoc comparison test, as specifically indicated. Differences were considered statistically significant when *p* < 0.05.

## 3. Results

### 3.1. DMF Is Well-Tolerated and Activates the Nrf2/HO-1 Pathway in HREC

**Figure 1:**
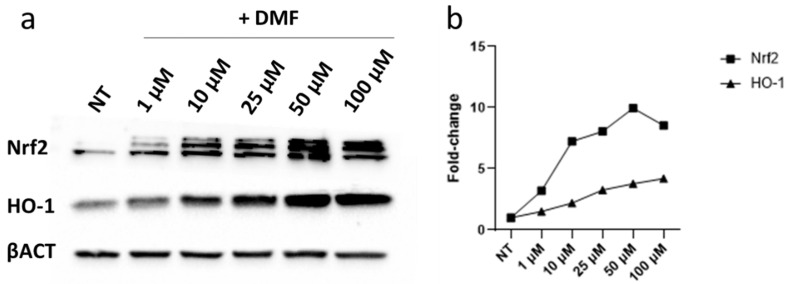
Immunoblotting analysis (**a**) of Nrf2 and HO-1 protein expression levels after DMF treatment. Cells were treated with an increasing concentration of DMF (i.e., 1 µM, 10 µM, 25 µM, 50 µM, and 100 µM) and analyzed 6 h after treatment (**b**) Densitometric analysis of Nrf-2 and HO-1 expression levels normalized on β-actin. On the Y-axis are reported the fold-change of treated cells compared with non-treated (NT) cells. On the X-axis is the concentration of DMF used.

We first tested through immunoblotting the expression of Nrf2 and HO-1 protein levels using increasing concentration of DMF (1 µM, 10 µM, 25 µM, 50 µM, and 100 µM) on HREC 6 h after treatment. As reported in Figure 1, a clear concentration-dependent increase in both Nrf2 and HO-1 was observed, starting from 10 µM of DMF. 

**Figure 2:**
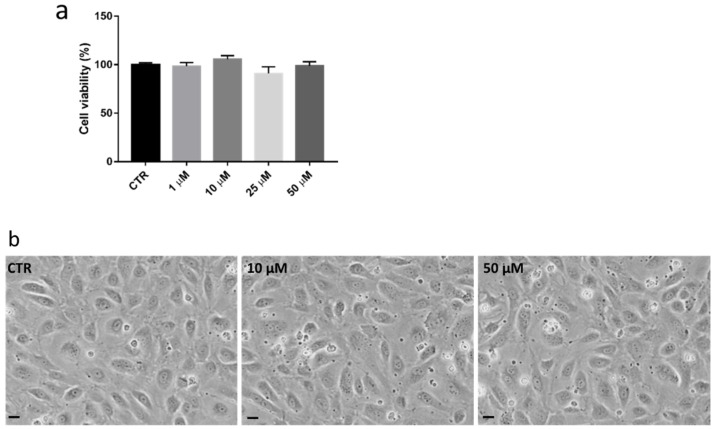
Analysis of cell viability and morphology of HREC after DMF treatment. (**a**) Cell viability with increasing concentrations of DMF (i.e., 1 µM, 10 µM, 25 µM, and 50 µM) or vehicle (CTR) for 72 h. Cell viability is expressed as percentage ± SEM. Dunnett’s multiple comparison test; N.S. *n* = 10–15. (**b**) Optical microscope observation of HREC cells non-treated (NT) and treated with DMF (e.g., 10 µM and 50 µM). Images are 20× magnification. Scale bars (=10 µm) are reported.

Subsequently, the effects of 72 h treatment with increasing amounts of DMF (1 µM, 10 µM, 25 µM and 50 µM) on HREC viability was tested using the PrestoBlue cell viability assay. As shown in Figure 2a, all the DMF concentrations ranging from 1 to 50 µM were well-tolerated and no morphological alterations were detected at optical microscope observations (Figure 2b). 

**Figure 3:**
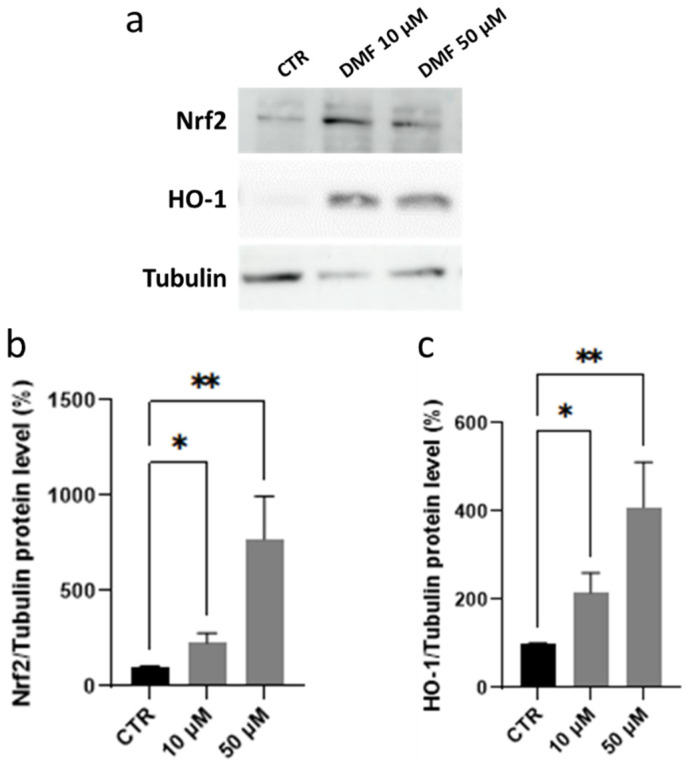
Evaluation and Western blotting representative gels of Nrf2 and HO-1 protein levels in total homogenates of HREC cells exposed to vehicle (CTR), 10 or 50 µM DMF for 6 h (**a**–**c**). α-Tubulin was used as a control to normalize the data. Results are expressed as percentages ± SEM. Dunn’s multiple comparisons test vs. CTR; * *p* < 0.05; ** *p* < 0.005.

We then performed explorative experiments to evaluate the potential Nrf2 pathway activation in HREC exposed to the same range of DMF concentrations for 6 h; this time was selected according to our experience of the DMF’s rapid action evidenced in other cellular models [31,32]. Among Nrf2-induced target genes, we focused on *HO-1* for the strong Nrf2-dependence [31] and for its relevant role in maintaining endothelial homeostasis [26,33]. HREC were exposed for 6 h to 10 µM and 50 µM of DMF, and the protein expression of Nrf2 and HO-1 was analyzed. As reported in Figure 3, immunoblotting analysis confirmed that a statistically significant increase of Nrf2 protein levels occurred in total homogenates of HREC after DMF treatment, and it was accompanied by an upregulation of HO-1 protein. The increase of both Nrf2 and HO-1 after treatment with DMF followed a concentration-dependent trend. Additional Western blotting experiments in HREC exposed for 24 h to either 10 µM or 50 µM DMF showed that the HO-1 protein level was much higher than that in control cells (+560% and +606%, for 10 µM and 50 µM, respectively; not shown), suggesting that a long-term exposure to DMF led to a sustained content of this stress defense factor. 

**Figure 4:**
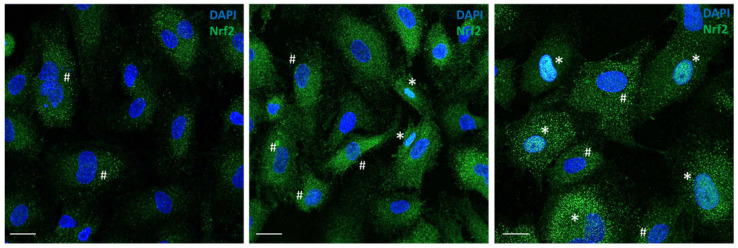
Evaluation of Nrf2 expression levels after DMF treatment (10 and 50 µM, 6 h after treatment). Nrf2 was labelled with AlexaFluor488. Nuclei were stained in DAPI. Scale bars (=10 μm) are reported. White asterisk indicates nuclear translocation of Nrf2 as diffuse signal; white hashtag indicates nuclear foci.

Nrf2 expression levels in HREC after DMF treatment were also investigated through immunofluorescence. As shown in Figure 4, at 6 h after DMF treatment a concentration-dependent increase in fluorescence associated with Nrf2 was observed. Particularly, HREC treated with 10 µM DMF showed an increased cytoplasmic fluorescent intensity and some events of nuclear translocation, mainly as nuclear foci; conversely, HREC treated with 50 µM DMF showed a marked increase in nuclear diffuse fluorescent signals.

**Figure 5:**
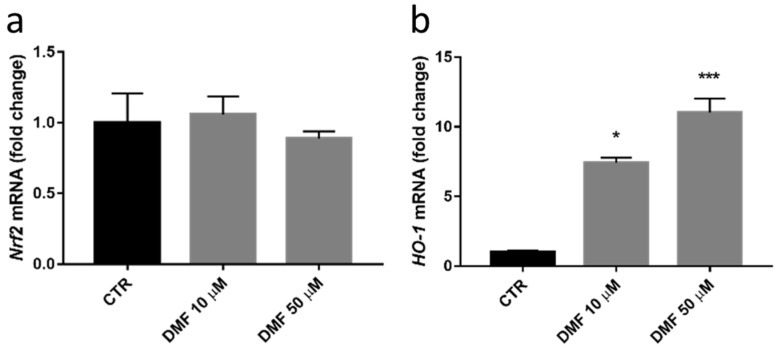
mRNA total levels in HREC cells exposed to vehicle (CTR), 10 µM or 50 µM DMF for 6 h. *Nrf2* (**a**) and *HO-1* (**b**) mRNA levels were examined by Real-Time quantitative PCR. *GAPDH *mRNA was used as a housekeeper to normalize the data (according to the formula 2^−^^ΔΔCt^). Results are expressed as fold change ± SEM. Dunn’s multiple comparisons test vs. CTR: * *p* < 0.05; *** *p* < 0.001; *n* = 3.

To evaluate whether Nrf2 and HO-1 protein increase were associated to upregulation of gene expression, both the transcripts were quantified by Real-Time quantitative PCR; these experiments evidenced that both concentrations of DMF strongly induced *HO-1 *mRNA expression but not *Nrf2* at 6 h (Figure 5a,b). Again, *HO-1* mRNA levels increased according to the concentration of DMF used. Considering these results, the minimum active concentration of DMF, 10 µM, was thus selected for the following experiments. 

### 3.2. DMF Protects HREC against Reactive Oxygen Species

**Figure 6:**
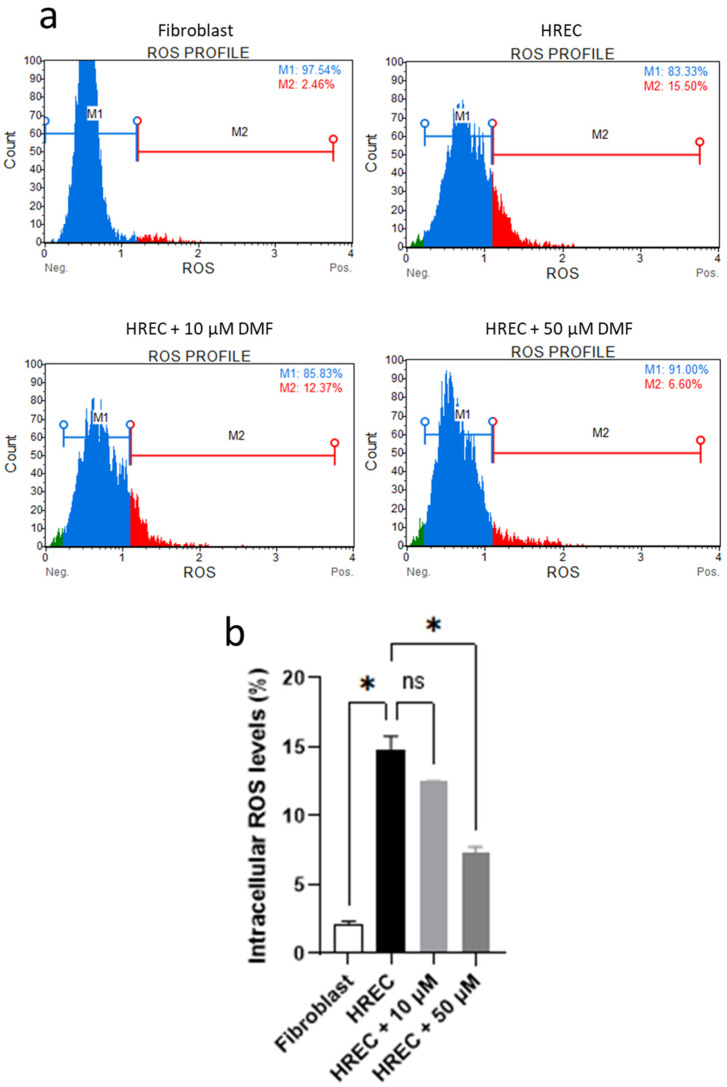
Intracellular ROS level analysis through flow cytometry. (**a**) Plots reporting the percentage of ROS(−) (blue) and ROS(+) (red) cells in the analyzed population. Events collected = 2000. (**b**) Histograms reporting the percentage of ROS(+) cells. * *p* < 0.05, Welch ANOVA. ns = not significant. Analysis was performed on three independent biological replicas.

The cytoprotective role of DMF in HREC was then investigated by means of cytofluorimetric analysis. Preliminarily, intracellular ROS levels in HREC were measured and compared to those present in human primary fibroblasts. Subsequently, the same analysis was performed at 6 h after treatment with 10 µM and 50 µM of DMF. As reported in Figure 6, HREC showed basal intracellular ROS levels statistically higher compared with human primary fibroblasts. Moreover, DMF treatment showed a decreasing trend in ROS levels, statistically significant only with 50 µM of DMF.

### 3.3. DMF Protects HREC under High Glucose Condition

**Figure 7:**
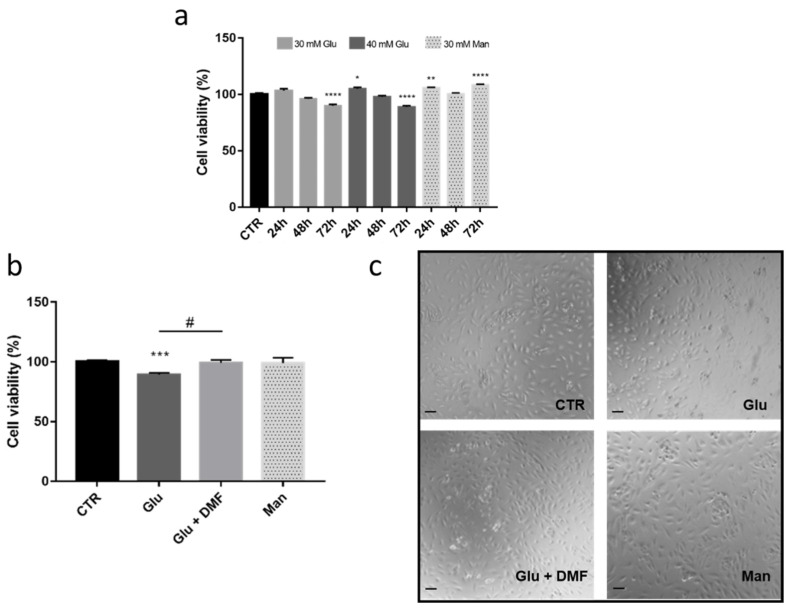
Cell viability of HREC under high glucose conditions. (**a**) Cell viability of HREC treated with vehicle (CTR), glucose (Glu; 30 and 40 mM) or mannitol (Man; 30 mM) for 24, 48, and 72 h. Viability is expressed as percentage ± SEM. Dunnett’s multiple comparisons test; * *p* < 0.05; ** *p* < 0.005; **** *p* < 0.0001 vs. CTR; *n* = 8–12. (**b**) Cell viability of HREC treated with vehicle (CTR), 30 mM mannitol (Man), or 30 mM glucose (Glu) ± DMF (10 µM) for 72 h. Viability is expressed as percentage ± SEM. Dunnett’s multiple comparisons test; *** *p* < 0.0001 vs. CTR; unpaired *t*-test; # *p* < 0.05 vs. glucose; *n* = 10–20. (**c**) Morphological evaluation of HREC with different treatments. Images were taken at 10× magnification.

*In vitro* studies report that endothelial cells are susceptible to high-glucose concentrations, although with notable differences among laboratories [34,35,36,37,38]. Before evaluating potential cytoprotective effects of DMF treatment in HREC cells under high glucose conditions, we first performed experiments to select the best time of exposure and concentrations of glucose leading to a significant cell viability impairment in HREC. According to the literature, HREC cells were treated with glucose (30 mM, 40 mM) for 24, 48, and 72 h, and cell viability tested using PrestoBlue assay. The mannitol (30 mM) was used as an osmotic control. Both 30 mM and 40 mM concentrations of glucose displayed a mild but statistically significant cytotoxicity in HREC after 72 h; mannitol did not affect the cell viability at 72 h, suggesting that the detrimental effect of high glucose on HREC survival is specifically related to this monosaccharide (Figure 7a). Our results are in agreement with previously published data [37]. HREC were thus concomitantly exposed to 30 mM glucose and 10 µM DMF and cell viability tested at 72 h to evaluate potential cytoprotective effects of DMF. The concentration of DMF was selected according to the previous results showing Nrf2 activation, with the aim of testing the effects of the lowest possible concentration of DMF. The mortality of 30 mM glucose-treated cells was confirmed at around 10%; statistical analyses showed no difference in the viability between control and cells treated with DMF under high glucose concentrations (100% and 97.2%, respectively) (Figure 7b). Compared only to their counterparts (89.3% viability in high glucose), cells exposed concomitantly to high glucose plus DMF showed statistically significant higher survival (+9.0%). By microscopic observation, no macroscopic changes among conditions were detected, although glucose-treated HREC presented slight morphological modifications and a lower number of viable cells (Figure 7c). Mannitol-treated cells looked like control cells; as well, DMF-treated HREC under high glucose appeared similar to control cells, suggesting that the presence of DMF confers cytoprotection from the hyperglucose concentration.

**Figure 8:**
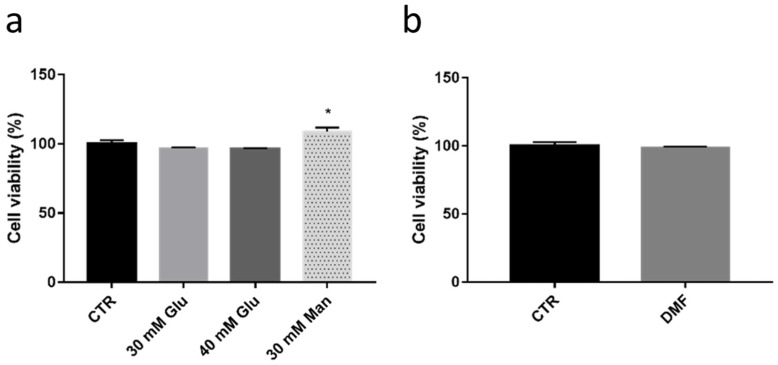
Viability evaluation of HREC after 6 days of treatment with glucose. (**a**) Viability of HREC cells treated with vehicle (CTR), glucose (Glu; 30 mM, 40 mM) or mannitol (Man; 30 mM) for 6 days. Viability is expressed as percentage ± SEM. Dunnett’s multiple comparisons test; * *p* < 0.05 vs. CTR; *n* = 8–12. (**b**) Cell viability of HREC cells treated with vehicle (CTR) or 10 µM DMF for 6 days. Viability is expressed as percentage ± SEM. Mann–Whitney test; N.S. *n* = 8–12.

In order to find a worsening impact on HREC viability, we then tested a longer treatment (6 days) with high glucose concentrations; in parallel, we exposed HREC to 10 µM DMF for 6 days to verify its good long-term tolerability or to show potential cytotoxicity in this cellular model. Surprisingly, 6 days with 30 mM and 40 mM concentrations of glucose determined no toxicity in HREC; mannitol induced a mild but statistically significant improvement of cell viability (+9.0% than CTR) (Figure 8a). The excellent tolerability of 10 µM DMF in HREC was confirmed at 6 days (Figure 8b). 

## 4. Discussion

Nrf2/Keap1/ARE signaling pathway is one of the most relevant defensive cellular systems against pro-oxidative and proinflammatory stress. Keap1 is the main inhibitor of Nrf2; in basal conditions, Keap1 binds to Nrf2 and directs it to proteasome for degradation, thus maintaining cellular Nrf2 at a low level. Following oxidation or modification of Keap1 by ROS or exogenous molecules, Keap1 dissociates from Nrf2, which migrates to the nucleus, inducing the expression of ARE-driven antioxidant, anti-inflammatory, and detoxifying genes, among which *HO-1* is one of the most relevant. Thus, Nrf2 pathway activation serves as either a physiological defensive response to a cellular insult or a preventive strategy induced by pharmacologically active molecules useful to increase the cellular defense against future potential stressors. Many Nrf2 activators, including DMF, have been tested in normal conditions or under stress stimuli, with the aim of improving or restoring the Nrf2 pathway, especially in contexts characterized by either increased oxidative stress and inflammation or a defective antioxidant/anti-inflammatory endogenous response.

The present *in vitro *study on DMF and the Nrf2/HO-1 pathway in human retinal endothelial cells relies on the following premises. First, the Nrf2 pathway represents an attractive therapeutic target for some retinal diseases [38,39]. Indeed, beside a number of systemic pathologies characterized by Nrf2 alterations, dysfunctions in the Nrf2 pathway have been related to various ocular diseases, such as diabetic retinopathy (DR), age-related macular degeneration, central retinal artery occlusion, uveitis, and glaucoma [40,41,42,43,44,45,46,47]. In the majority of these contexts, abnormalities at a vascular level are detectable and contribute to the onset/progression of the disease as either primary or secondary pathogenic factors. Second, and strictly related to the previous statement, a proper Nrf2 functioning is relevant for endothelial homeostasis and vasoprotection; the evidence from several studies strongly suggests that Nrf2 alteration in endothelial cells is associated with ageing and various age-related pathologies, such as ischemia, cardiovascular diseases, and DR [23,48,49,50]. 

DR is a diabetes-associated, multifactorial progressive disease of the retina characterized by a complex pathogenesis that involves different cells, factors, and molecules. DR affects both neural and vascular districts of the retina, leading to progressive visual impairment and even blindness [23,51]. Perturbation of Nrf2 has been suggested to complicate DR. Involvement of the Nrf2 pathway in DR is supported by *in vivo *studies showing that, among others, a high glucose state in diabetes impairs Nrf2 activation and decreases Nrf2-target genes in the retina [43,52]. Moreover, human studies show subnormal Nrf2 signaling in the retina from DR donors [52] and that patients with recent onset type 2 diabetes present significantly lower Nrf2 circulatory levels than healthy subjects [53]. The underlying mechanisms of low Nfr2 expression in diabetes remain largely unknown. Overall, these data indicate that targeting Nrf2 in the retinal endothelium may be useful and of great interest for the therapy of DR and beyond.

This research study aimed to evaluate DMF as an activator of the Nrf2 pathway in HREC cells and to test its potential cytoprotective effects under a DR-related stress condition. We found that long-term treatments with DMF, also at high concentrations (up to 50 µM), are optimally tolerated in HREC. DMF (10 µM for 6 h) is able to induce an increase of Nrf2 total protein levels, accompanied downstream by an upregulation of HO-1 expression, at both the mRNA and protein level. This evidence was also supported by immunofluorescence experiments showing Nrf2 nuclear translocation. The DMF-induced 8-fold increase of *HO-1* mRNA is an index of Nrf2 transcriptional activation; the result is consonant with the concomitant robust upregulation of HO-1 protein detected at the same time and suggests a *de novo* HO-1 protein synthesis. No change in *Nrf2* transcript level is instead detected after 6 h of 10 µM DMF in HREC. Higher concentration of DMF (i.e., 50 µM) led to a statistically significant increase of both Nrf2 and HO-1 protein levels. As observed for the 10 µM concentration, no changes in *Nrf2* mRNA levels were detected; conversely, a statistically significant increase in *HO-1* mRNA levels was observed, even higher compared with the 10 µM concentration. Altogether, these results suggest that DMF is able to induce Nrf2 activation in a concentration-dependent manner, as well as an increase of the protein level of the downstream effector HO-1. The absence of *Nrf2* mRNA induction might be explained with the hypothesis that, in the tested condition, the Nrf2 protein increase is more probably due to a lower Nrf2 degradation than a new gene expression. Interestingly, treatment with 10 µM DMF also did not show any toxic effect after 6 days, strengthening the safety of this molecule. 

The beneficial effects of DMF in counteracting ROS formation were then investigated in HREC by measuring the intracellular ROS levels by flow cytometry. DMF treatment induces a concentration-dependent decrease of intracellular ROS levels, thus suggesting a cytoprotective role in the maintenance of the redox status. This is of particular relevance in HREC, which showed higher basal ROS levels compared to those detected in human primary fibroblasts. These findings thus confirm that endothelial cells are more prone to accumulate ROS due to their high metabolic demand [29] and reinforce the rationale of DMF and other antioxidant molecules as preventive strategy against oxidative stress, especially in the retina, one of the highest oxygen-consuming tissues of the body.

The potential protective role of DMF in the context of hyperglucose conditions was also studied. As reported in the literature, this condition leads to oxidative stress as well as mitochondria and endoplasmic reticulum (ER) stress [54,55,56]. Specifically, we found that 30 mM glucose for 72 h impairs the HREC viability by 10% and that 10 µM DMF confers cytoprotection, restoring the viability to control levels, as also suggested by optical microscope observations. Conversely, cell viability was not affected after 6 days of hyperglucose. The proposed hypothesis is that HREC adapted to the high glucose in our experimental setting, due to the possible transient cytotoxic effects of this condition. This hypothesis will be investigated in future studies. Interestingly, in the literature, there is a high variability in both experimental settings and results regarding the status of HREC/endothelial cells under high glucose concentrations (≥25 mM). In agreement with our data, other *in vitro* studies showed no relevant changes at 24 h [34,35]; however, at the same time of exposure, Jin et al. [36] recently reported that 30 mM glucose in HREC induces proliferation (about +20%). These discrepancies in both the acute and chronic hyper-glucose-induced effects may be related to different experimental settings among laboratories, including the source and passage of HREC and the method used to assess cell survival, with the majority of *in vitro* studies performing the MTT assay. Regarding this latter test, we used the PrestoBlue test, considered the most reliable method to evaluate viability in human endothelial cells [57]. 

Nrf2 short-term pharmacological activation ameliorates vascular dysfunction in aged rats and in different pathological human vasculatures [58]. Several Nrf2 activators are known to counteract diabetes-induced endothelial dysfunctions, but few are currently in clinical trials [59]. Therefore, the ability of DMF to activate the Nrf2/HO-1 pathway in the retinal vascular endothelium may have relevant therapeutic implications. Strength points of DMF are surely the well-characterized, good pharmacological and toxicological profiles and the longstanding clinical use in psoriasis and relapsing-remitting multiple sclerosis [16,60]. DMF is a small molecule (MW 144.14), potentially easy to be delivered to the retina by either topical or systemic treatment; in accordance with this, a case report refers clinical benefits after oral assumption of DMF for 36 months in a patient with edema at the retinal level [20]. 

Importantly, to our knowledge, this *in vitro* study is the first systematic investigation on DMF and the Nrf2 pathway performed in human primary endothelial cells from the retina. Indeed, previous *in vitro* studies on DMF effects in primary endothelial cells were carried out in human umbilical vascular endothelial cells (HUVEC) [24] and human brain microvascular endothelial cells (HBMVEC) [61]. By far, the potential role of DMF and its derivatives as protective agents for retinal diseases has received little attention [62,63]; our findings give a new impulse to this perspective and go further, suggesting that DMF can be repurposed in eye pathologies involving alterations of the endothelium and, more extensively, in ocular diseases characterized by oxidative stress and inflammation. 

## Data Availability

The datasets used and analyzed in this study are available from the corresponding author upon reasonable request.
